# Supplementary material for: Evaluating the implementation of the Reproductive Life Plan in disadvantaged communities: A mixed-methods study using the i-PARIHS framework
Source: PLoS One. 2020 Sep 11;15(9):e0236712. doi: 10.1371/journal.pone.0236712 (PMC7485818; doi:10.1371/journal.pone.0236712)
Supplement: S1 Appendix — (PDF) [file pone.0236712.s001.pdf]

The Reproductive Life Plan tool

Questions to ask:

Do you want to have any (more) children at any time in your future?

IF YES:

a. How many children would you like to have?
b. How long would you like to wait until you and your partner becomes pregnant?
c. What family planning method do you plan to use until you and your partner are ready to become pregnant?

IF NO:

a. What family planning method will you use to avoid pregnancy?
b. People's plans change. Is it possible you and your partner could ever decide to become pregnant?

Action Steps

Once your client has a plan–encourage her to take action, depending on the answer the following can be discussed:

Yes, I want children soon

- ☐ How to improve financial situation
- ☐ Advice on birth spacing if less than two years since last baby
- ☐ Refer to clinic to:
  - Test and start antiretroviral therapy
  - Adhere to antiretroviral therapy
  - Check if woman has illness* that can affect pregnancy
- ☐ Multivitamin, iron supplements and folic acid
- ☐ Avoid alcohol, drugs and smoking
- ☐ Healthy food and physical activity
- ☐ Age
- ☐ Antenatal care immediately when pregnant
- ☐ Exclusive breastfeeding after birth
- ☐ Tetanus vaccination

Yes, I want children sometime in the future

- ☐ Family planning
- ☐ Keep a normal weight to be healthy and to be able to conceive and have a healthy pregnancy in the future
- ☐ Condom to protect from STIs to be able to conceive in the future

No, I don't want children

- ☐ Family planning

*e.g. epilepsy, diabetes, disease in thyroid gland, high blood pressure, tuberculosis, kidney disease, heart and vascular disease, mental disease and asthma.
