# Supplementary material for: Evaluating the implementation of the Reproductive Life Plan in disadvantaged communities: A mixed-methods study using the i-PARIHS framework
Source: PLoS One. 2020 Sep 11;15(9):e0236712. doi: 10.1371/journal.pone.0236712 (PMC7485818; doi:10.1371/journal.pone.0236712)
Supplement: S2 Appendix — (PDF) [file pone.0236712.s002.pdf]

# Discussion guide

Both positive and negative aspects will be probed, and when needed, follow-up questions will be asked to clarify statements. The numbers indicate main questions and the letters indicate follow-up questions.

## Focus group discussion at baseline in January 2018

**Themes:**

- The RLP-intervention
- Family planning discussions
- Partner involvement

**Questions:**

1. What do you think about using the RLP-tool? **[General opinion RLP]**
    a. Imagine that you would use this tool when meeting a client. How do you think that would work?
2. How can the RLP-intervention be adapted to your context? Is it possible to use it as it is now? **[Improvement/adaption]**
    a. If you were about to use this with one of your clients – how would you put the questions? Do you think it would work, if not – why?
3. What factors affect family – and pregnancy planning discussions? **[Factors FP/RLP discussions]**
    a. In what situations is it hard to discuss these topics? Is there anything in the society, culture or religion that you think affect the discussions in your country?
4. What challenges do you face when discussing family planning with your clients? **[Challenges/doubts]**
    a. In what situations and with what type of patients are these discussions more challenging? Do you know why it is like that?
5. What is the men's role in family- and pregnancy planning? **[Partner involvement]**
    a. Do men need to be involved in these discussions? How can they be involved? What challenges are there to do this?

## Focus group discussion at follow-up in May 2018

**Themes:**

- Experiences from using the RLP-tool
- Developing the RLP-tool further
- The future of the RLP-tool in this context

**Questions will be asked on:**

1. What is your overall experience of using the RLP-tool? **[Overall experience]**
    a. In what way was it …?
    b. How did you introduce the RLP into the counselling? How did you pose the question?
    c. How was it to use it if the client said no/yes/maybe?
2. Can you recall any specific experiences when using the RLP-tool? **[Specific experience]**
    a. Any situations where you felt it worked or did not work?
    b. You told me some women are lazy (group 1,2), how did the RLP work with them?
    c. Women from Mozambique (group 1)? With teenagers? Was it possible to involve partners?
3. What do you think about using the RLP-materials (RLP-checklist and the report sheet)? **[RLP-material]**
    a. Can you describe any aspects of the material that was difficult to understand
    b. Would it be useful to document the woman's answer?
4. How did your clients react when using the RLP? **[Client's reaction]**
    a. How did you feel when using it? How did the men/partners react?
    b. What happened at the clinic? Can they fulfill their goals?
5. How can the RLP-tool be improved? **[Improvement after]**
    a. What issues are most important to make women achieve their reproductive goals and how can those be highlighted?
6. What do you think about using the RLP in the future? **[RLP in future]**
    a. What challenges, if any, can you see about using the RLP as a routine in your work?
7. Last time we discussed unplanned pregnancies. How does the RLP relate to unplanned pregnancies? How can the RLP contribute to this matter? **[RLP and unplanned pregnancy]**
8. What is your opinion about the *feasibility* of the RLP-instrument? Please describe. **[feasibility]**
9. How much *effort* did it take for you to use RLP? **[effort]**
10. What knowledge and skills were important for you to have in order to have successful RLP conversations? **[knowledge and skills]**
11. Was RLP highlighted during supervision? How was it? **[knowledge about own practice]**
12. In what ways can using the RLP improve pregnancy and family planning for your clients? Do you believe that using the RLP will improve…? **[expected outcome]**
13. How would you like to use RLP in the future? What training or continuing education do you prefer? **[learning style]**
14. Do you think you are capable of using it in the future? **[self-efficacy]**

15. How did the referral to the clinic work? Did any of you refer patients to the clinic? (e.g. to the antenatal care if they want to conceive) **[Referral processes]**
16. What resources are necessary for using RLP successfully? Which did you have? Did you have all the necessary resources to use the RLP? **[Availability of necessary resources]**
17. How can communicate RLP in the group? What did you think about using the WhatsApp group? **[communication and influence]**
18. Would you like to receive feedback on the use of RLP in the field? **[Monitoring and feedback]**
