# Supplementary material for: Evaluating the implementation of the Reproductive Life Plan in disadvantaged communities: A mixed-methods study using the i-PARIHS framework
Source: PLoS One. 2020 Sep 11;15(9):e0236712. doi: 10.1371/journal.pone.0236712 (PMC7485818; doi:10.1371/journal.pone.0236712)
Supplement: Attachment — Submitted filename: Response to reviewers.docx [file pone.0236712.s004.docx]

Response to reviewers

**Reviewer #1:**

Dear editor, thanks for asking me to review this manuscript again. The authors have done really a good job in revising their manuscript. They addressed majority of the comments and the manuscript reads much better now. I really enjoyed reading it to the end. I think the international readers will benefit from reading this article. I have only minor comments that I would like to share

**Authors’ response:** Thank you for constructive comments and we are happy that you think the manuscript has improved. We have responded to the comments point-by-point below.

1. Methods section at the beginning is very important and may need to be supplemented with a little more [brief] details before detailed description comes later on. There is a figure that explains to the reader the intended plan for the study, but again this is introduced later.

**Authors’ response:** Thank you, we have added a few more details in the description and moved figure 1 ‘Study procedures and timeline’ to the first paragraph in the methods section (page 7, line 87-91). We agree this increases readability.

2. In line 110 [starting from “We…”] there was an abrupt move from describing the setting and the population to describing the sample and recruitment. I suggest separating this into a new section called sample and recruitment and add more details on this. Did you recruited the whole population to participate in the study, or you have selected part? How was the selection? Etc.

**Authors’ response:** Good suggestion, we have separated this information into a new section (page 8, line 112-119). 53 MMs were eligible and we estimated 29 informants would be sufficient. The selection of MMs was based on the geographical area where they are assigned to work, as we wanted to have representatives from each region. Discussions on data saturation were held continually and we chose to not include more MMs as we deemed data saturation was reached in the primary analysis of the results.

3. For me the use of the questionnaire in this study has added very little value, the rich discussions that took place in the group interviews has covered in more depth the areas that was asked about in the questionnaire. I would remove this part, in my opinion it will make the description of the study methods and reporting of the results more straightforward and easier. But I will leave this decision to the authors.

**Authors’ response:** We wish to keep the questionnaire as we think it adds credibility to the findings (as discussed on lines 562-566) and as the use of the NoMAD questionnaire has not been reported from any low- or middle-income setting before.

4. In the limitation section the authors have described their roles etc, I don’t see this as necessary.

**Authors’ response:** We wish to keep this information as it is required in the COREQ (Consolidated criteria for Reporting Qualitative research) Checklist.

**Reviewer #3:**

Overall, the revised paper has addressed the questions I had. The only comment I have is to include in the limitation section that the study did not record the number of RLP implementations each MN attempted to deliver and actually delivered during the intervention period. These data may provide some insights on MN's qualitative feedback.

**Authors’ response:** Thank you for valuable feedback and we are happy that you feel we have addressed the questions you had. We monitored the number of RLP interventions by using report sheets, where the MMs filled out how many clients they had approached. We did not intend to use this information in the research mainly, rather as a way of facilitate implementation of the intervention, but we agree that this would have added valued when interpreting the results. There was a problem with lack of copies of the report sheets and of losing the report sheets. Therefore, one of the MMs suggested that this reporting would be included in the ordinary patient records and this have now been amended. As you suggest, we have added this as a limitation in the methods section (page 28, line 567-568).
